# Supplementary figures and images for: A ‘double-edged’ role for type-5 metabotropic glutamate receptors in pain disclosed by light-sensitive drugs
Source: eLife. 2024 Aug 22;13:e94931. doi: 10.7554/eLife.94931 (PMC11341090; doi:10.7554/eLife.94931)

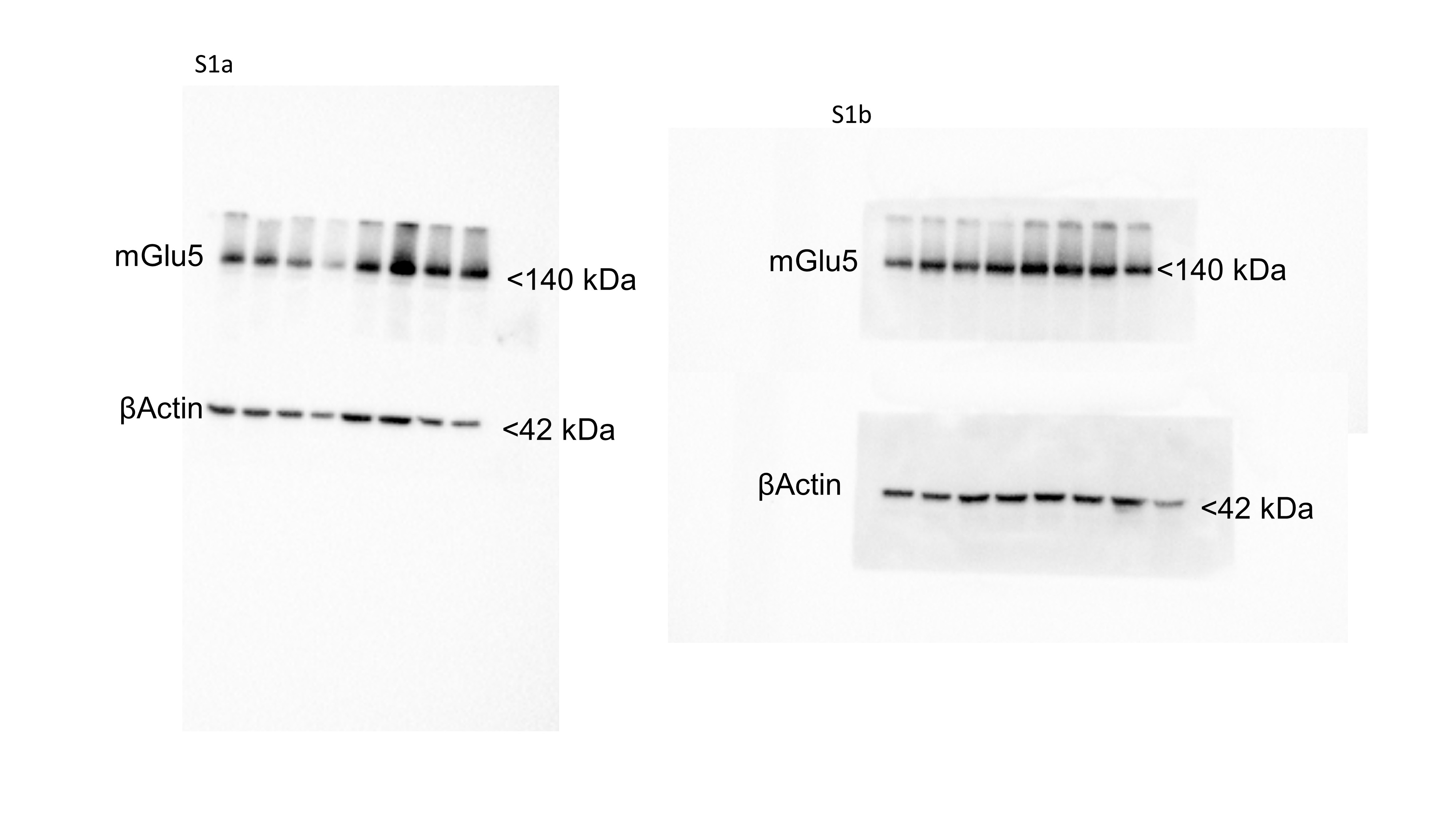

Supplement: Figure 1—figure supplement 1—source data 1. [file elife-94931-fig1-figsupp1-data1.zip › Figure 1-figure supplement 1-source data 2.TIF]

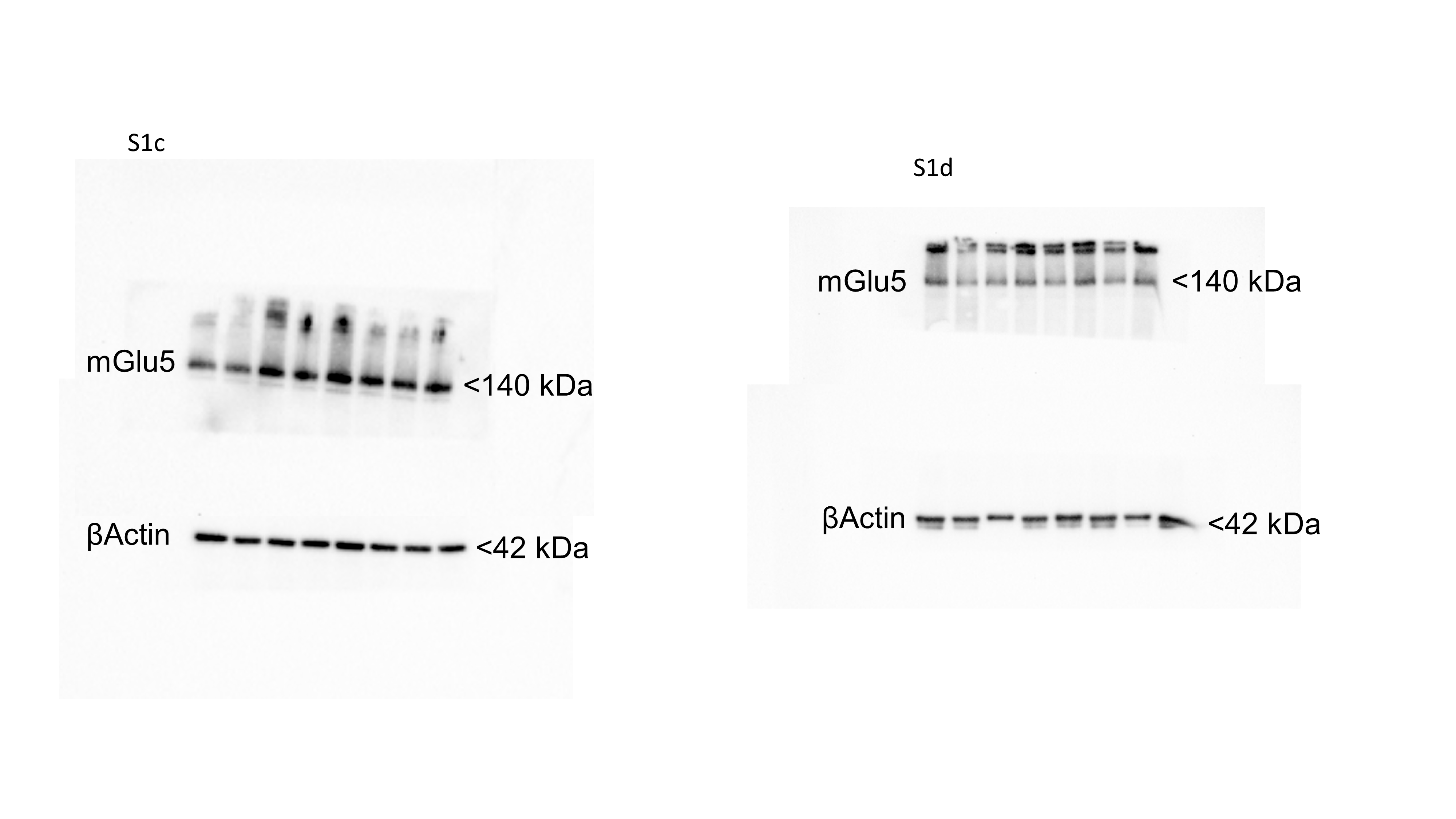

Supplement: Figure 1—figure supplement 1—source data 1. [file elife-94931-fig1-figsupp1-data1.zip › Figure 1-figure supplement 1-source data 3.TIF]

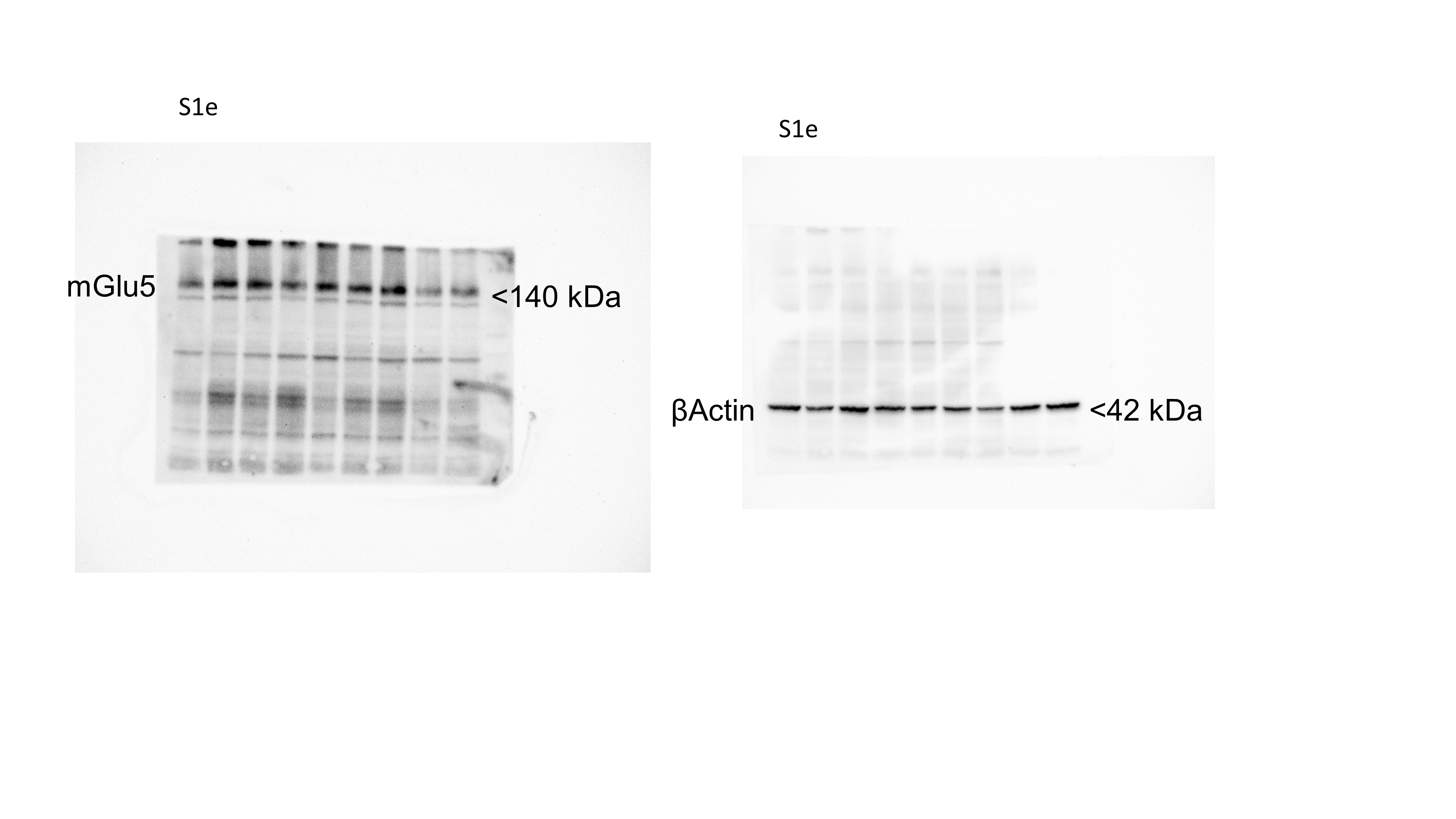

Supplement: Figure 1—figure supplement 1—source data 1. [file elife-94931-fig1-figsupp1-data1.zip › Figure 1-figure supplement 1-source data 4.TIF]
