# Supplementary material for: A ‘double-edged’ role for type-5 metabotropic glutamate receptors in pain disclosed by light-sensitive drugs
Source: eLife. 2024 Aug 22;13:e94931. doi: 10.7554/eLife.94931 (PMC11341090; doi:10.7554/eLife.94931)
Supplement: Supplementary file 1. [file elife-94931-supp1.docx]

| **GROUPS** | **Cohen's d** |
| --- | --- |
| Pre-drug vs Alloswitch-1 | (20.2 - 26.16) ⁄ 6.266087 = **0.951152** |
| Alloswithc-1 vs Violet light | (29.4 - 20.2) ⁄ 9.562531 = **0.962088** |
| Violet light vs Green light | (29.4 - 23.35) ⁄ 12.979856 = **0.466107** |

**Table 1. Effect size of optical modulation in prelimbic cortex on RVM activity, according to Cohen’s d calculation from t-tests.**
